# Exosome nanovesicles: biomarkers and new strategies for treatment of human diseases

**DOI:** 10.1002/mco2.660

**Published:** 2024-07-15

**Authors:** Chuan Xu, Chaoyang Jiang, Zhihui Li, Hui Gao, Jing Xian, Wenyan Guo, Dan He, Xingchen Peng, Daijun Zhou, Dong Li

**Affiliations:** ^1^ Department of Oncology The General Hospital of Western Theater Command Chengdu China; ^2^ Department of Oncology The Second Affiliated Hospital of Chengdu Medical College China National Nuclear Corporation 416 Hospital Chengdu Sichuan China; ^3^ Department of Biotherapy Cancer Center West China Hospital Sichuan University Chengdu Sichuan China

**Keywords:** drug delivery, exosome, immunotherapy, liquid biopsy, targeted therapies

## Abstract

Exosomes are nanoscale vesicles of cellular origin. One of the main characteristics of exosomes is their ability to carry a wide range of biomolecules from their parental cells, which are important mediators of intercellular communication and play an important role in physiological and pathological processes. Exosomes have the advantages of biocompatibility, low immunogenicity, and wide biodistribution. As researchers’ understanding of exosomes has increased, various strategies have been proposed for their use in diagnosing and treating diseases. Here, we provide an overview of the biogenesis and composition of exosomes, describe the relationship between exosomes and disease progression, and focus on the use of exosomes as biomarkers for early screening, disease monitoring, and guiding therapy in refractory diseases such as tumors and neurodegenerative diseases. We also summarize the current applications of exosomes, especially engineered exosomes, for efficient drug delivery, targeted therapies, gene therapies, and immune vaccines. Finally, the current challenges and potential research directions for the clinical application of exosomes are also discussed. In conclusion, exosomes, as an emerging molecule that can be used in the diagnosis and treatment of diseases, combined with multidisciplinary innovative solutions, will play an important role in clinical applications.

## Graphical abstract

• Updated review of recent exosomes in liquid biopsy and disease treatment applications

• Recent advances in the use of exosomes as biomarkers in disease applications

• Therapies and efficacy of engineered exosome applications

• Impact of exosomes on precision medicine and personalized treatment

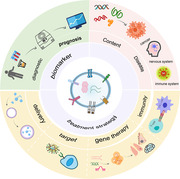

## 1 INTRODUCTION

Extracellular vehicles (EVs) are lipid bilayer vesicles released from the cell and have been recognized as crucial entities that play an important role in intercellular, intertissue, and interorgan communicatio.^1^, ^2^, ^3^ Exosomes are tiny vesicles 30−150 nm^4^, ^5^ in diameter, a subclass of EVs, which can be secreted by a variety of cells,^4^, ^6^, ^7^, ^8^, ^9^, ^10^ and are enriched in specific biologically active molecules such as proteins, lipids, nucleic acids, depending on the source.^11^, ^12^, ^13^ Since the role of exosomes in intercellular communication was first discovered by Sebastian Amigorena's research group in 1998, it has been recognized as a new mode of intercellular communication as research has progressed. Exosomes can directly activate intracellular pathways in target cells by binding to specific receptor ligands,^14^ and can also stimulate target cells through membrane fusion or through endocytosis/phagocytosis to deliver a large amount of information,^15^ such as proteins, lipids, and nucleic acids, to the receptor cells, completing the signaling in the receptor cells,^16^, ^17^ thus participating in the regulation of physiological and pathological processes such as immune response, antigen presentation, and tumor invasion.^18^

Exosomes can be isolated from a variety of body fluids, including bronchoalveolar fluid,^19^ cerebrospinal fluid,^20^ blood,^21^ urine,^22^, ^23^ saliva,^21^, ^24^ breast milk,^21^ semen,^25^ amniotic fluid,^26^ synovial fluid,^13^ as well as fluid from diseased lesions including ascites and pleural fluid,^27^ and can be subjected to multicomponent analyses.^28^ Excitingly, exosomes are mediators of intercellular communication, and their secretion and content change as the disease progresses.^29^ Most notably, they can circulate steadily in body fluids and can be used for dynamic tracking.^30^ These properties determine the use of exosomes for liquid biopsies, which have been demonstrated in a wide range of diseases. Especially in tumor research, exosomes can be used as specific cancer biomarkers and diagnostic biomarkers for tumor diagnosis, treatment, efficacy assessment, and so on.^31^, ^32^, ^33^, ^34^, ^35^ Similarly, studies have proved that exosomes play an important role as biomarkers in neurodegenerative diseases,^36^, ^37^, ^38^ cardiovascular,^39^, ^40^, ^41^ hepatic,^42^, ^43^ infectious diseases,^44^, ^45^, ^46^ and autoimmune disease.^47^, ^48^, ^49^, ^50^ It is currently recognized as the most promising biomarker in the field of liquid biopsy.^51^, ^52^, ^53^

Despite the advances in contemporary medicine, there are still serious challenges in the diagnosis and treatment of many diseases. It is well known that early diagnosis of diseases before they reach the clinical stage is very important for effective prevention, treatment, and significant improvement of prognosis.^54^ However, there are many limitations to early diagnosis, such as invasiveness, low sensitivity, high cost, and low acceptance.^55^, ^56^, ^57^, ^58^ In the treatment of some intractable diseases (tumors, neuropsychiatric disorders, autoimmune diseases, and infectious diseases), despite the addition of many new drugs, the therapeutic effect has been seriously affected by the problems of blood–brain barrier (BBB) obstruction, drug tolerance, and poor drug targeting.^59^

Exosomes, as excellent key mediators of cell communication, can not only be used as biomarkers for early diagnosis and monitoring of diseases, but also play an active role in drug delivery, targeted therapy, and immune regulation^60^ due to their biocompatibility and protective bilayer lipid structure to protect genetic cargo from degradation, coupled with reduced immunogenicity. Because their small size and membrane composition allow them to cross the BBB,^61^ they also shed new light on neurological and psychiatric disorders.^62^, ^63^

In this review, our main aim is to explore the potential of exosomes in disease management. We introduces the key advances of exosomes in liquid biopsy, drug delivery, targeted therapy, immune regulation, and gene therapy. Revealed the achievements of extracellular vesicles in clinical research, pointed out the challenges faced in clinical translation, and finally explored the impact of extracellular vesicles in precision medicine and personalized therapy.

## 2 EXOSOME BIOGENESIS AND COMPOSITION

### 2.1 Biogenesis

**FIGURE 1:**
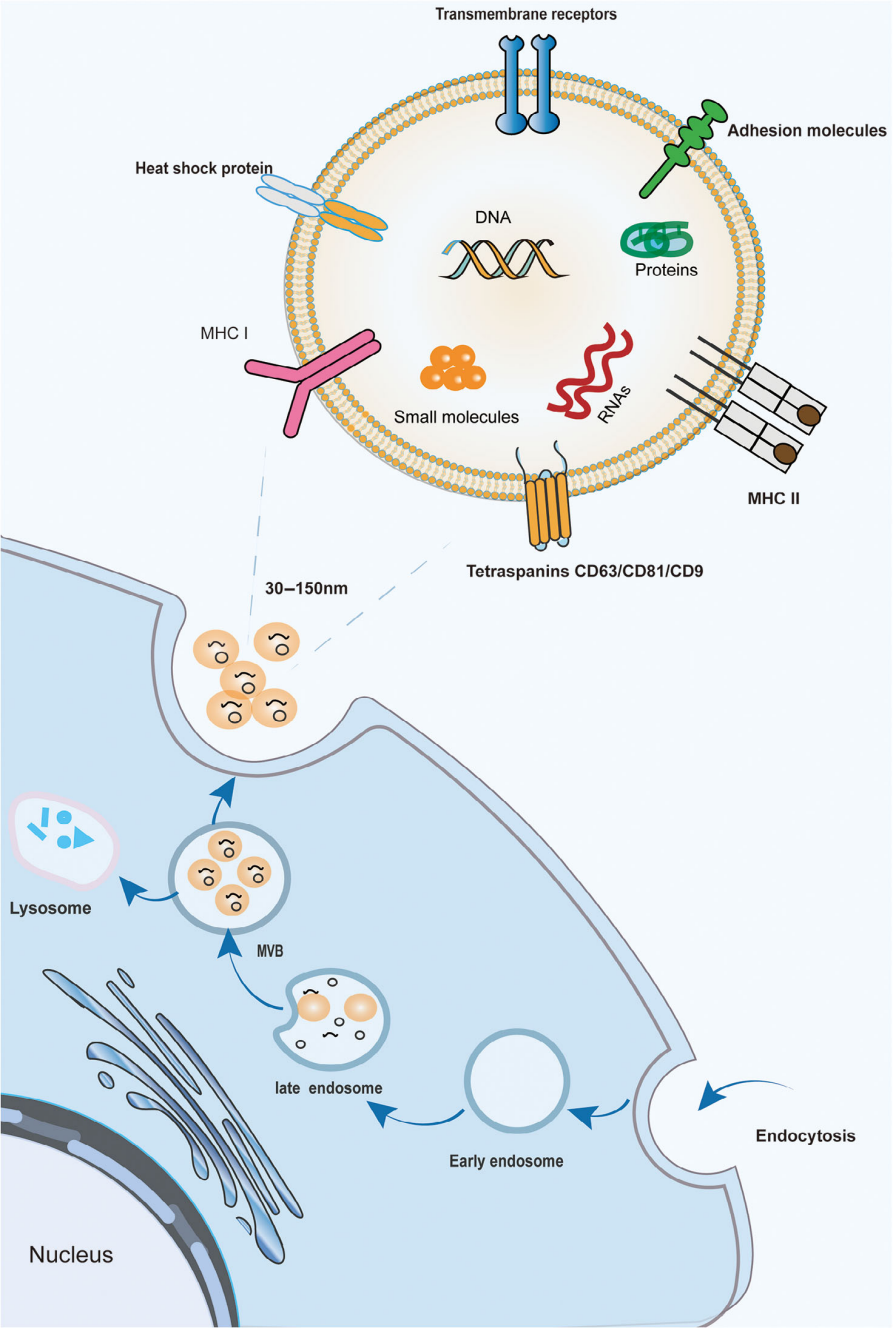
Biogenesis and composition of exosomes. Exosome formation begins with endocytosis of the cytoplasmic membrane, from which the cell membrane folds inward to form early endosomes, which mature into late endosomes, and eventually into multivesicular bodies (MVB). Eventually, MVB either undergoes degradation or fuses with the plasma membrane to release exosomes containing specific cargoes. The composition of exosomes is very complex and is mainly divided into proteins, lipids, and nucleic acids (by Adobe Illustrator). MHC, major histocompatibility complex; MVB, multivesicular bodies.

Exosome formation begins with endocytosis of the cytoplasmic membrane in three main stages.^64^, ^65^ (1) Early endosomes are formed by the inward folding of the cell membrane. They are essentially lipid vesicles. (2) Multivesicular bodies (MVB) are formed. Early sorting endosomes progress to late sorting endosomes, which are membrane invaginated to form smaller nano‐vesicles (30–150 nm)^66^ called intraluminal vesicles (ILVs), which restrict membrane endocytosis to form MVBs containing multiple ILVs. (3) The MVB fuses with the plasma membrane and releases ILVs with specific cargoes called exosomes^64^ (Figure 1). MVB formation is essential for exosome biogenesis and can occur through two pathways, the endosomal sorting complex required for transport (ESCRT)‐dependent pathway and the ESCRT‐nondependent pathway.^67^ ESCRT is involved in the development of the MVB and ILV, which contains four protein complexes, ESCRT‐0, ‐I, ‐II, and ‐III and vacuolar protein sorting related protein 4.^67^ The nonclassical pathway includes specific lipid molecule‐driven mechanisms^68^ and tetraspanin‐mediated drive mechanism.^69^, ^70^ Specific lipid molecules that are involved in the regulation of MVB formation include mainly ceramide, phosphatidic acid, and sphingosine‐1‐phosphate,^68^, ^71^ which participate in inward budding and lead to ILV formation through lipid raft‐mediated and amino acid‐dependent pathways.^72^, ^73^ It has been found that the tetraspanin involved in secretion regulation (CD63, CD9, CD82, CD81, etc.) can polymerize with each other to dynamically recruit multiple transmembrane protein molecules or signaling molecules on the cell surface or organelle membranes to form specific multimeric complexes.^74^, ^75^, ^76^ This “tetraspanin web” can effectively promote the sorting and enrichment of other cargo proteins on endosomal membranes and the formation of ILVs.^77^ The fact that these two pathways for the biogenesis of exosomes are not entirely separate,^78^ they seem to work synergistically, and the diversity of exocytosis depends on the different machinery, as well as the cell types involved.^77^

### 2.2 Composition

Exosomes induce intracellular signaling by delivering their contents to receptor cells via either receptor–ligand binding or plasma membrane fusion. The composition of exosomes is therefore an important factor in determining the effectiveness of their action. Lipids are one of the most essential components of exosomes, as the lipid bilayer composition determines their potential to be internalized by recipient cells and utilized for therapeutic development.^79^ Several lipid molecules in exosomes, including sphingolipids, cholesterol, and phosphatidylserine, are located in the membrane.^80^ Sphingolipids and cholesterol play a major role in the stability of the exosome phospholipid bilayer,^81^, ^82^ whereas phosphatidylserine promotes the fusion of exosomes with target cell membranes and acts as a signaling molecule in signal transduction.^83^

Based on the proteomic analysis, more than 4400 relevant proteins have been selectively enriched in specific exosomes,^84^ which are classified into five main groups,^85^ including (1) MVB manufacturing proteins (Alix, Tsg, tetraspanins, clathrinid) and transport proteins(annexins, flotillin, Rab); (2) heat shock proteins (HSP; HSP70, HS9083); (3) signaling molecules (HIF‐1α, PI3K, ADP‐ribosylation factor 1 (ARF1), β‐catenin); (4) cytoskeletal proteins (actin, tubulin, cofilin, profiling, myosin, vimentin, fibronectin, meosin, keratins, talin); (5) various enzymes (GAPDH, ATPase, pgk1). Some of the aforementioned proteins participate in the normal physiological activities of exosomes, whereas other proteins mediate interactions between exosomes and recipient cells.

In addition, EVs also contain large amounts of nucleic acids (e.g., mRNAs, micro RNAs, noncoding RNAs, piRNA, and DNA).^64^ These functional RNAs can affect the transcriptome of recipient cells,^86^, ^87^ with miRNAs being the most extensively studied. They can inhibit the expression or translation of target genes after transcription, disrupt the stability of mRNA and inhibit its translation, regulate the expression of target genes^88^ in different types of cells, and participate in important biological processes such as cell proliferation, differentiation, apoptosis, and metabolism.^89^ Kahlert et al.^90^ identified large fragments (>10 kb) of genomic double‐stranded DNA containing all chromosomes found in exosomes and can be used to detect mutated DNA in serum, which can be used for cancer prediction, treatment, and therapy resistance.

The composition of exosomes depends to some extent on source cell type and can also be affected by different cell conditions or treatments.^91^ In addition, the exosomes released by a cell line may be highly heterogeneous, which determines the functional properties of the exosomes.^92^ This ability of exosomes to deliver their contents to receptor cells produces a bi‐directional effect, which can be involved in the diagnosis and treatment of disease, and is also closely related to certain diseases' development and progression.

### 2.3 The role of exosomal cargo in disease

Exosomes act as important messengers for the exchange of substances between cells,^93^, ^94^ facilitating the transfer of important information between cells and regulating cellular functions through the transmission of contents.^95^ Detection of specific molecules carried by exosomes provides a new way to understand the mechanism of disease occurrence and development. In recent years the contents of exosomes are associated with the development of a variety of diseases, especially in the field of cancer,^78^ which has received sustained attention.

#### 2.3.1 Cancer

**FIGURE 2:**
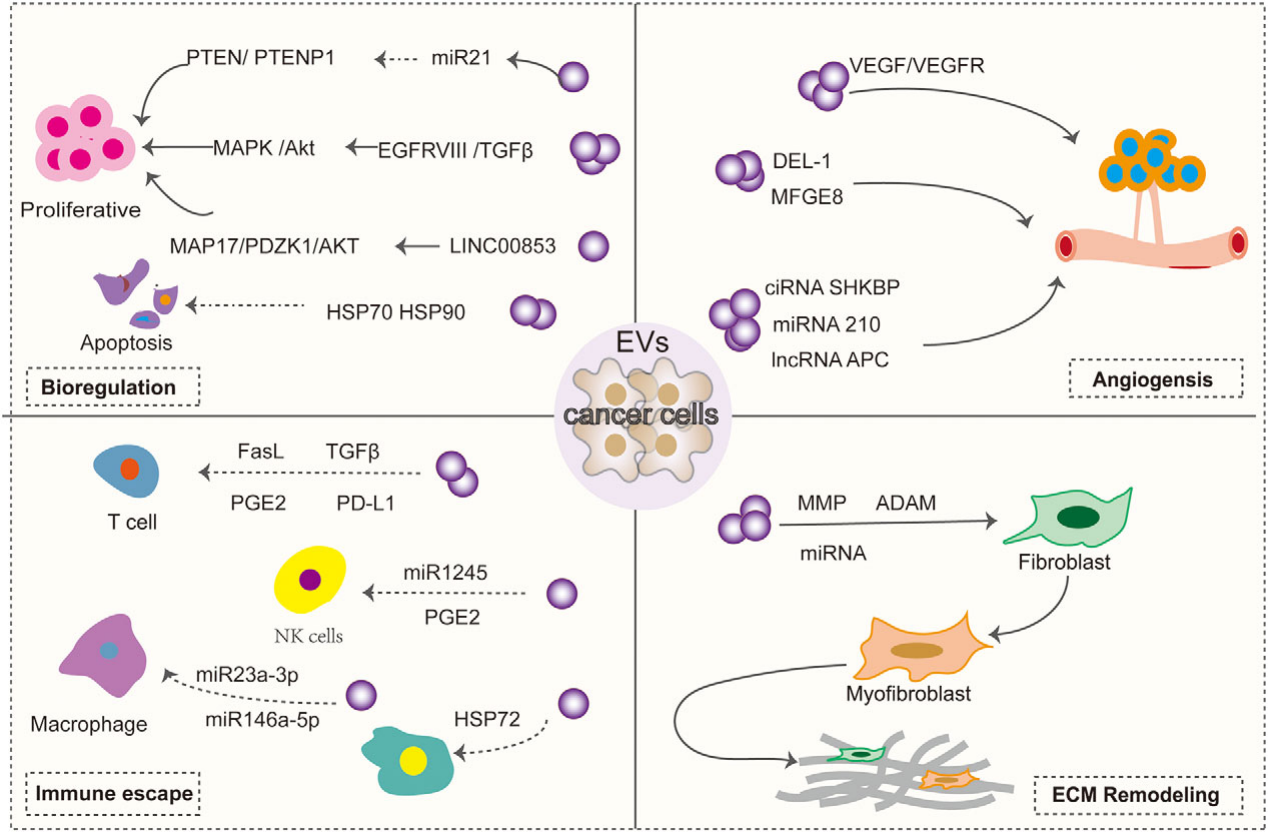
Role of exosomal cargoes in tumorigenesis and development. Exosomes of tumor cell origin can be involved in bioregulation, proangiogenesis, matrix remodeling, and immune evasion of tumor cells by delivering the proteins, nucleic acids, lipids, and other contents they carry (by Adobe Illustrator). PTEN, phosphatase and tensin homolog; PGE2, prostaglandin E2; NK‐cells, natural killer cells; MMP, matrix metalloproteinase; ADAM, a disintegrin and metallo‐proteinases; DEL‐1, developmentalendotheliallocus‐1; MFGE8, milk fat globule eGF factor 8; VEGF, vascular endothelial growth factor; APC, adenomatous polyposis coli.

Exosomes regulate tumor and host cell functions by docking with receptor cells and delivering the proteins, nucleic acids, lipids, and other contents they carry. Exosomes derived from tumor cells can participate in the bioregulation, proangiogenesis, matrix remodeling and immune evasion of tumor cells (Figure 2). All these mechanisms suggest that exosomes are closely related to tumors and help promote tumor cell development and metastasis.

##### Biomodulation of tumor cells

High epidermal growth factor receptor (EGFR) expression is strongly associated with neurogliomas. EVs derived from brain tumors have been identified to contain both EGFR and EGFRvIII (constitutively active mutant receptors). In U373 glioma cells EGFRvIII can be transferred via EV to distant cells lacking EGFRvIII expression, while activating downstream signaling pathways in these cells and cell transformation is induced.^96^, ^97^ In addition, ligands such as epidermal growth factor(EGF), transforming growth factor alpha(TGFα), and two‐regulator proteins have been found in the EV of breast and colorectal cancer cell lines. These EV‐containing ligands can stimulate recipient cells and induce proliferation.^98^ Similarly, HCC cells produce the exosome miR‐21, which regulates the expression of the tumor suppressor genes PTEN and PTENp1 through a variety of mechanisms, thereby affecting the proliferation of HCC cells.^99^ HCC cell‐derived exosomes are also enriched in Golgi membrane protein 1, which activates the GSK‐3β/MMPs signaling axis in the recipient cells and accelerates cell proliferation and migration.^100^ Survivin in tumor exosomes, as well as the heat shock proteins HSP70 and HSP90, have also been shown to inhibit apoptosis and increase cell proliferation and invasion, stimulate the tumor microenvironment, and promote the growth and spread of primary tumors.^98^, ^101^ Recently, LINC00853 in gastric cancer tissues was found to promote cell proliferation, invasion, and migration by regulating the MAP17/PDZK1/AKT pathway.^98^ MiR‐92a, a cancer‐associated fibroblasts(CAF)‐derived exosome, acted as a facilitator of breast cancer cell migration and invasion by reducing G3BP2.^102^

##### Inducing angiogenesis

Angiogenesis is the formation of new blood vessels from preexisting ones.^103^ Abnormal angiogenesis is a feature of several pathologies including cancer. Tumor EVs promote angiogenesis by promoting vascular endothelial generation response. It has been found that tumor‐derived EVs contain a variety of proteins associated with angiogenesis. For example, EV derived from A431 squamous carcinoma cells can transfer oncogenic EGFR to endothelial cells, activate the MAPK and AKT pathways, as well as increase the expression of endogenous vascular endothelial growth factor (VEGF) and induce angiogenesis.^104^ Glioblastoma‐derived exosomes containing proangiogenic proteins, angiopoietins, VEGF, IL‐6, and IL‐8 stimulate increases in endothelial tube length and angiogenic proteins and stimulate in vitro angiogenesis in the brain microvascular endothelial tubule formation assay.^105^ In particular, EVs released in hypoxic environments increase the activation of ERK1/2 MAPK, PI3K/AKT, and FAK pathways in recipient endothelial cells, leading to more endothelial cell sprouting. Breast cancer tumor‐derived EV promotes VEGF receptors and tumor angiogenesis via VEGF90K.^106^ Hegmans et al.^107^ analyzed EV secreted by several mesothelioma cells by MALDI‐TOF MS and identified DEL‐1 in EV, which is thought to play a role in angiogenesis.^108^ MFGE8, a major component of EV from immature dendritic cells and tumor cells, promotes VEGF‐induced cell survival and thus induces angiogenesis.^109^, ^110^ Similarly, proteins associated with angiogenesis have been found in exosomes derived from hepatocellular carcinoma, ovarian carcinoma, and nasopharyngeal carcinoma.

The study also highlights that noncoding RNAs in exosomes play a key role in tumor angiogenesis. Circ‐RNAs (circ‐SHKBP,^111^ circRNA‐100338^112^) in tumor exosomes can act as ceRNAs to inhibit miR‐877‐3p, thereby increasing VEGFA expression,^111^ and can also regulate angiogenesis by interacting with the RNA‐binding protein NOVA2.^112^ LncRNAs(lncRNA‐H19,^113^ lncRNA‐APC,^114^ lncRNA‐MALAT1,^115^ lncRNA‐TUG,^116^ lncRNA‐p21,^117^ lncRNA‐GAS5,^118^ lncRNA‐AHIF,^119^, ^120^ lncRNA‐HOTAIR,^121^ lncRNA‐CCAT2,^122^ lncRNA‐POU3F3^123^) promote the expression of key proangiogenic genes and proteins by acting as ceRNAs or binding to mRNAs. A variety of EV miRNAs are involved in tumor angiogenesis by regulating endothelial cell activity (e.g., miR‐23a,^124^ miR‐25‐3p,^125^ miR‐26a,^126^ miR‐182‐5p,^127^ miR‐221,^128^ miR21,^129^ miR‐210,^130^ miR‐9^131^). MiRNAs also can promote angiogenesis by inhibiting FOXO1 expression.^132^

##### Immune escape

Immune escape is one of the major hallmarks of cancer. During tumorigenesis and progression, EV suppresses immunity through a variety of mechanisms, a process mainly mediated by exosomes released from tumors.^133^ Tumor‐derived EVs can carry immunosuppressive cargoes that prevent immune cells from attacking circulating tumor cells. Among these, protein programmed death ligand 1 (PD‐L1) is the predominant cargo. For example, binds to T cells and induces immunosuppression^134^; inhibits the activation of NK and CD8+ cells through miR1245, which downregulates the expression of the NKG2D receptor^135^, ^136^; and it can also stimulate prostaglandin E2 secretion by increasing the myeloid‐derived suppressor cell (MDSC),^137^ which inhibits NK cells as well as CD4 and CD8 lymphocytes.^138^ HMGB1 in tumor‐derived exosomes also promotes immune evasion in HCC by promoting the expression of TIM‐1 and regulating the growth of B cells.^139^ Tumor exosomes contain nucleic acids that are also involved in immune evasion. For example, the thyroid‐derived exosome miR‐519e‐5p can aid tumor immune escape from distant organs by inhibiting the Notch signaling pathway.^140^ Exosomal circCCAR1 released by HCC cells contributes to immunosuppression by facilitating CD8 + T‐cell dysfunction in HCC.^141^ Exosomal lncRNA‐TUC339, circTMEM181, miR‐23a‐3p, and miR‐146a‐5p, components of the tumor‐derived exosome, promote M2 macrophage polarization, thereby impeding the efficiency of CD8+Tcells.^142^ In addition, some EVs produced by cells with immunosuppressive properties are involved in immune evasion, such as Hsp72, the content of MDSC‐derived EV, activates signaling and transcriptional activator 3 (Stat3), which stimulates MDSC immunosuppressive function.^143^, ^144^

##### Extracellular matrix remodeling

Abnormal deposition or loss of extracellular matrix (ECM) components may bring about many changes in the microenvironment that are associated with disease progression. Tumor‐derived EVs enriched with proteases, including a variety of MMP (MMP14, MMP9, MMP13, MMP1, and MMP3), as well as the ADAM family of disintegrins, can regulate the ECM, thereby favoring tumor progression, as described in detail by Nawaz et al.^145^ Tumor exosomes can also remodel ECM by recruiting and reprogramming normal fibroblasts into cancer‐associated fibroblasts (CAF). It has been shown that TGF‐β expressed on the surface of cancer‐derived EVs triggers the TGF‐β/SMAD3 signaling pathway in recipient fibroblasts, which induces the expression of a myofibroblast‐like phenotype (α‐SMA, FGF2)^146^; EV also generates CAFs through a nonclassical fibronectin‐dependent pathway.^147^ Furthermore, tumor exosomes contain miRNAs that reprogram recipient cells, which produce CAF.^148^

#### 2.3.2 Neurological diseases

Research has shown that EVs transfer proteins and genomic materials between neurons, which is crucial for the progression of neurodegenerative diseases. These vesicles contribute to the propagation of these neurotoxic substances, leading to synaptic dysfunction and widespread neuronal damage.^60^ EV derived from neurons contains amyloid‐β peptides and tau proteins.^149^ During the development of Alzheimer's disease (AD), pathological tau protein is distributed in exosomes released by microglia.^150^ Insoluble aggregates of tau proteins can induce ROS production in a calcium‐dependent manner through activation of NADPH oxidase, leading to neuronal damage. Exosomes carrying α‐synuclein disseminate and accumulate between neurons, leading to degeneration and death of dopamine neurons.^151^ Studies have also highlighted that exosomal miRNAs are closely associated with the development of neurodegenerative diseases.^152^ MiRNAs play a key role in the upregulation of PD‐associated proteins such as α‐synuclein, tau proteins, DJ‐1, and so on.^153^ EV is also able to translocate miR‐21‐5P from the neuron to the microglial cell, leading to microglial M1 polarization and excessive release of proinflammatory cytokines, thereby aggravate neurological damage.^154^

#### 2.3.3 Immune system diseases

EV contents also play a pathogenic role in autoimmune diseases. EV carries the peptide‐major histocompatibility complex (MHC), which presents antigens to T cells and participates in the formation of immune complexes,^155^ inducing an inflammatory response by stimulating the production of tumor necrosis factor (TNF) by macrophages.^148^ LncRNAs in exosomes that mediate NF‐κB and Wnt/β‐catenin signaling pathways are involved in the pathogenesis of rheumatoid arthritis (RA).^156^ Additionally, cytokines carried by EVs, such as interleukin‐6 (IL‐6) and TNF‐α, contribute to the inflammatory environment.^157^ Exosomal contents miRNAs are also involved in disease pathogenesis by regulating the secretion of key pathogenic cytokines^158^ and the direction of immune cell differentiation.^159^

Similarly, it has been proven that the proteins and nucleic acids carried by EVs play a pathogenic role in infectious diseases, cardiovascular diseases,^160^ blood diseases, and more.^60^

## 3 EXOSOME BIOMARKERS

Exosomes from different cell types have different signaling molecules because of their unique contents. With a full understanding of the relationship between exosomal contents and disease occurrence, exosomes are considered potential therapeutic targets and biomarkers.^161^ Moreover, it stably exists in various body fluids and has the advantages of high sensitivity, low invasiveness, and easy access to materials compared with other tests,^17^ which can help in the early diagnosis of diseases, detection of diseases, and evaluation of therapeutic effects (Table 1).^32^, ^162^

**TABLE 1:** Applications of exosome biomarkers.

Diseases	Biomarker	Appliance	References
**Cancer**			
Pancreatic	miR‐1293	Prognostic biomarker	^163^
Liver	miR‐638	Prognostic biomarker	^164^
Ovarian	miRNA‐205	Diagnostic biomarker	^165^
	miR‐1290	Diagnostic biomarker	^166^
	ZNF587B	Diagnostic biomarker	^167^
Intestinal tract	miR‐3127‐5p	Diagnostic biomarker	^168^
	miR‐1470	Diagnostic biomarker	^169^
Lung	circ_0061407 circ_0008103	Diagnostic biomarker	^170^
Cholangiocarcinoma	circ‐0000367	Diagnostic and monitoring biomarker	^171^
	circ‐0021647		
	circ‐0000288		
Glioblastoma	EGFR EGFRvIII	Diagnostic	^172^
	miR‐151‐3p	Diagnostic	^173^
Breast	miR‐21	Diagnostic	^174^
	miR‐27a		
	miR‐375		
**Immune disease**			
IgA nephropathy	miR‐451a	Prognosis biomarker	^175^
Rheumatoid arthritis	lncRNA:ENST00000433825.1	Prognosis biomarker	^176^
	miR‐885‐5p	Diagnostic	^47^
	miR‐6894‐3p		
	miR‐1268a		
Idiopathic pulmonary fibrosis	hsa_circ_0044226、	Diagnostic	^177^
	hsa_circ_0004099		
	hsa_circ_0008898		
SLE	miR‐451a	Diagnostic	^178^
**Cardiovascular**			
Acute myocardial infarction	circ_0001558	Diagnostic	^179^
	PTEN	Diagnostic	^180^
	miR‐126		
	miR‐21		
Coronary heart disease	circ_0001360	Diagnostic	^181^
	circ_0000038		
**Neurodegenerative**			
AD	miRNA‐125b	Diagnostic	^182^
	miRNA‐451a		
	miR‐135a	Diagnostic	^183^
PD	miR‐331‐5p	Diagnostic	^184^
	miR‐505		
	miR‐153	Diagnostic	^185^
	miR‐223		
**Communicable disease**			
Tuberculosis	miR‐484	Diagnostic	^46^
	miR‐96		
	miR‐425		
	miR‐let7e‐5p	Treatment response monitoring	^186^
	M. tuberculosis‐derived RNA		
HIV	miR‐378	Treatment response	^187^
	miR‐630		
	miR‐378		
	miR‐630		

Abbreviations: AD, Alzheimer's disease; EGFR, epidermal growth factor receptor; PD, Parkinson's disease; PTEN, phosphatase and tensin homolog deleted on chromosome ten; SLE, systemic lupus erythematosus..

### 3.1 Cancer

As is well known, early diagnosis of tumors is particularly important in cancer treatment, closely related to the success rate and prognosis of patients.^188^ Exosomes contain a large amount of information about cancer cells,^189^ which is closely related to cell proliferation, angiogenesis, metastasis, and immune response regulation processes. They can be used as biomarkers for liquid biopsy to reflect in vivo tumor activity. At present, research has confirmed that exosomes have become an important tool for early cancer screening and detection.^190^ The most extensively studied area is miRNA, and Jafari et al.^191^ provided a detailed review of the application of extracellular vesicle miRNA as a biomarker in early cancer diagnosis. zNF587B plays a crucial role in early ovarian cancer detection.^167^

In addition, exosomes circulate in body fluids and can provide a wealth of information about the status of the cancer,^192^ offering valuable insights at all stages of disease progression. A meta‐analysis shows that up‐ and downregulated circulating exomiRs may serve as a valid indicator of inferior survival outcomes in patients with gastrointestinal malignancies.^193^ In fact, serum‐specific exomiRs have also been associated with the prognosis of several cancers. Circulating miR‐122 expression is upregulated in BC, non‐small cell lung cancer (NSCLC), associated with distant metastasis and reduced OS and PFS, which are considered prognostic factors.^194^ Low levels of miR320 were associated with advanced stage, LNM, and poorer OS in BC patients,^195^ as well as serum exosomal miR‐940 levels reflecting the presence of lymph node metastases and EGFR type 2 status, which may be a biomarker for metastasis in breast cancer.^196^ Elevated ExomiR‐301a was associated with the recurrence of gliomas.^197^ Recently, it has also been found that the exosome miR‐5100 in hypoxic head and neck squamous cell carcinoma (HNSCC) promotes CAF activation by coordinating the QKI/AKT/STAT3 axis, which further facilitates the metastasis of HNSCC. miR‐5100 has been associated with the malignant progression of HNSCC and may be a potential biomarker and therapeutic target of HNSCC.^198^

Other nucleic acids and proteins of exosome contents may serve as biomarkers for cancer surveillance. Serum exosomes circ_0061407 and circ_0008103 may be involved in NSCLC progression by interacting with microRNAs and proteins, and may serve as developmental and potential diagnostic biomarkers for NSCLC.^170^ Plasma exosome‐derived Cx43 has been shown to be a possible prospective prognostic indicator of 5‐year OS and 5‐year DFS in melanoma patients.^199^ Meanwhile, cancer cell‐derived exosomes have also demonstrated a promising role in monitoring resistance to chemotherapy and targeted drugs.^200^, ^201^

In summary, the use of exosomal cargoes as biomarkers can track early changes in cancer, progression, and drug efficacy over time.

### 3.2 Neurodegenerative diseases

The ability of exosomes to easily cross the BBB makes them very excellent biomarkers for determining CNS disease and response to therapy. Differential expression of specific proteins, miRNAs, and cirRNAs contained in exosomes offers potential for diagnosis and treatment of neurodegenerative diseases.^202^ Studies have demonstrated the consistency of blood and cerebrospinal fluid exosomes as biomarkers to answer questions about brain‐related diseases,^203^, ^204^ for early detection of disease and monitoring of disease progression in a noninvasive manner.

Pathologic changes in neurodegenerative diseases begin 10−20 years before clinical manifestations,^205^ and it is particularly important to find early diagnostic biomarkers with high specificity. Exosomes, as traceable molecules, are now hot biomarkers for early detection and monitoring of progression. A case–control study confirms that levels of P‐S96‐tau, P‐T181‐tau, and Aβ1‐42 in neurogenic blood exosomal extracts predict the onset of AD up to 10 years prior to the clinical onset of the disease.^206^ This was followed by studies confirming that blood neuroendocrine synaptic proteins, such as GAP43, SNAP25, neurogranin, and synaptotagmin 1, can be used as reliable biomarkers to predict AD 5−7 years before cognitive decline.^207^ In the Parkinson's study, investigators found that pathologically soluble a‐syn detected in plasma‐derived neuronal extracellular vesicles distinguished patients with Parkinson's disease from healthy controls.^208^ AD L1EV‐Synaptic nucleoprotein measurements in combination with specific precursor markers (such as olfactory or cognitive impairment, rapid eye movement sleep behavior disorder, or glucosinolates genetic status), can be used to help stratify individuals at high risk for Parkinson's disease and related Lewy body disorders.^209^

Exosomes also play an important role in monitoring disease progression and determining severity. The SNAP25 (synaptosome‐associated protein of 25 kDa) and synaptophysin‐I binding protein in neuron‐derived exosomes can predict the development of AD.^210^ Complement proteins (C1q, C3b, and C3d) and complement regulatory proteins (CD59, CD46, decay‐accelerating factor, and complement receptor type 1) in astrocyte‐derived exosomes (ADEs) can be further elevated or decreased as the disease progresses, and therefore, measurement of the levels of complement proteins in the exosomes can predict AD progression.^210^ In addition, reduced levels of specific excitatory synaptic proteins contained in neuron‐derived exosomes in plasma may indicate a correlation between the degree of cognitive loss and the severity of AD progression.^211^ The ratio of α‐syn oligomers to α‐syn in plasma exosomes of PD patients correlates with the severity of PD,^212^ and the mitochondrial respiration‐related markers they contain may be potential biomarkers for assessing the effect of restoration of mitochondrial function in PD recovery.^213^

### 3.3 Cardiovascular disease

Exosome‐borne elements also show great clinical potential in the diagnosis and prognostic monitoring of cardiovascular diseases.^214^ Exosomes containing noncoding RNAs (miRNAs,^215^, ^216^, ^217^ lncRNAs,^218^ cirRNAs^179^, ^181^) have been suggested to function as coronary artery disease (CAD) biomarkers and to be part of their specific therapeutic strategies, especially miRNAs. Levels of miR‐942‐5p, miR‐149‐5p, and miR‐32‐5p in blood exosomes correlate with atherosclerosis formation and may serve as a novel diagnostic and prognostic indicator of the disease.^219^ MiRNAs are also highly specific for cardiac injury,^220^ with higher sensitivity and specificity than the detection of troponin T. They can be used as indicators for the early diagnosis of myocardial infarction, and these elevated miRNAs (miR‐192, miR‐194, miR‐34a) may be predictors of the development of heart failure (HF) after acute myocardial infarction.^221^ Many miRNAs are involved in the diagnosis/prognosis of HF^222^ and have the ability to differentiate aspects of CAD patients with HF from those without HF.^223^ To further facilitate detection, the investigators also monitored circulating stained EVs by specifically staining atherosclerotic plaque (AS) foam cell‐derived exosomes directly in the circulation by fluorescence spectrometry or zymography, enabling early detection and real‐time differentiation of lesion vulnerability during AS progression, thereby facilitating effective CVD management.^224^

Recent studies report plasma exosomes to identify three early warning biomarkers of OST4, PKIG, and RPL23 associated with the genetic characterization of cardiac exosomes and used to create noninvasive and direct columnar graphic models to accurately predict the risk of CAD deterioration.^225^ Li et al.^226^ also found that increased expression of plasma exosome‐derived cysteine‐rich protein 61 (Cyr61) was associated with the acute coronary syndrome (ACS) and could be used in the diagnosis and prognosis of ACS. In addition, observation of urinary UMOD levels can be used as an early diagnostic biomarker for myocardial infarction.^227^ Of course, exosomes have been shown to be excellent in predicting early recurrence of atrial fibrillation,^228^ detection of cardiotoxicity of anticancer drugs,^229^ and diagnosis of aortic dissection.^41^

### 3.4 Infectious diseases

In the early stage of HIV infection, EVs can carry HIV viral RNA and proteins,^230^ and their content can change with HIV infection and treatment,^231^ so analyzing the size, number and molecular content of EVs can help in early diagnosis and observation of disease progression and treatment efficacy.^232^, ^233^ MiRNA‐21 is a promising and valuable prognostic biomarker in elite controller patients infected with HIV‐1 virus.^234^ Neuron‐derived EV isolated from plasma can be an excellent source of liquid biopsy biomarkers for identifying neurocognitive deficits in HIV patients.^204^, ^235^ HMGB1, NF‐L, and A proteins in exosomes are potential HIV‐1 prognostic biomarkers,^236^ alpha‐2‐macroglobulin, properdin, and hemopexin may serve as potential markers of substance abuse in HIV‐infected individuals who are regular smokers and alcohol drinkers.^237^ In addition, urinary EV collected from HIV‐positive individuals may provide a good source of HIV biomarkers, including p24.^238^

In viral hepatitis, exosomes released from infected hepatocytes contain diagnostically significant amounts of viral RNA, core proteins, envelope proteins, and DNA.^236^ Researchers have shown that exosomes can store HBV DNA and exosome‐based diagnostics exosomes may help predict the likelihood of HBV relapse.^239^ When a patient's serum HBV‐DNA is negative, exosomal HBV‐DNA accurately reflects the level of HBV replication in the body and allows for tracking of treatment.^240^ Characterization of Mycobacterium exocytosis RNA from infected macrophages reveals unique mRNA fingerprints and preferential exocytosis markers for host‐mRNA–miRNA and TB RNA in Mycobacterium tuberculosis infection.^241^ Distinctive expression patterns of exosomal miRNAs (miR‐148a‐3p, miR‐150‐5p, and miR‐451a) in pleural effusions from benign wounds and tuberculosis could serve as potential biomarkers for tuberculosis pathology.^242^ In addition, by analyzing the expression patterns of various genes, the investigators clarified the role of exosomal RNA biomarkers in the differential diagnosis of latent TB infection and active TB disease.^243^

The study of COVID‐19 also showed that exosomes in the blood can serve as potential biomarkers for predicting infection outcomes. Exosome surface CD142 is a platelet tissue factor (an extrinsic pathway for activation of the coagulation cascade) that has been associated with morbidity and mortality in COVID‐19 and is considered to be the most unique exosome surface protein for determining prognosis in COVID‐19.^244^, ^245^ Measuring the surface‐specific C‐reactive protein (CRP), alpha 1‐acidic glycoproteins (A1AG1 and A1AG2), and CXCL7 protein levels of exosomes can also help identify critically ill COVID‐19 patients.^246^

### 3.5 Autoimmune diseases

Exosomes containing immune‐related substances have also made a mark as biomarkers for autoimmune diseases. Circulating exosomes purified from the serum of patients with systemic lupus erythematosus (SLE) are immunologically active, and their relative levels correlate with disease activity in SLE patients, with dysregulation of exosomal miRNAs being the most studied.^247^ The circulating exosomes miR‐21 and miR‐155 are upregulated in SLE and are potential biomarkers for the diagnosis of SLE and LN.^248^ Circulating exosome miR‐451a levels were significantly lower in the group without renal damage SLE than in the group with renal damage, and can be used to assess SLE activity and detect the risk of renal injury.^178^

Researchers note that exosomal miRNAs in SLE patients are predominantly enriched in urine.^249^ Urinary exosome miR‐146a negatively correlates with traditional nephritis biomarkers (C3, C4) and proteinuria and is a predictor of SLE disease activity.^250^ In patients with LN, miR‐29c levels in urinary exosomes are highly sensitive and specific for early assessment of renal fibrosis and prediction of LN severity.^251^ MiR‐26a levels in urinary exosomes from LN patients compared with healthy controls that correlated positively with urinary protein levels, suggesting its potential use as a predictive biomarker of podocyte injury.^252^ Urinary exosomal miRNAs can also predict the response to treatment in LN. Urinary exosomal miR‐31, miR‐107, and miR‐135b‐5p levels were elevated in the post‐treatment response group, suggesting that they can be used as early biomarkers of LN prognosis.^253^

In recent years, various extracellular vesicle‐related biomarkers have been developed for RA. Among circulating exosomes, miR‐335‐5p, miR‐486‐5p, SRSF4,^254^ miR‐25‐3p,^255^, ^256^ and DPYSL3 and PSME1 proteins^257^ can be used for early diagnosis. Exosomal lncRNAs have also been extensively studied in RA due to their advantages in regulating gene expression. Research has shown that lncRNAs in RA plasma exosomes have characteristic expression profiles and potential for diagnostic biomarkers and therapeutic targets.^258^ The high expression level of HOTAIR in the blood monocyte exosomes of RA patients suggests that it may be a potential biomarker for the diagnosis of RA.^259^ NEAT1 and PCGEM1 have the potential to help clinicians better diagnose RA.^156^ In terms of disease monitoring, exosomal miR‐548a‐3p is negatively correlated with key laboratory indicators related to RA disease activity, which may be a predictive factor for RA disease activity.^260^ After tofacitinib administration, RA‐HCV patients had a significant increase in has‐mir‐155‐5p and has‐mir‐122‐5p compared with patients without HCV, which may be potential biomarkers of treatment efficacy in HCV RA patients.^261^ Recently, in the analysis of exosomes derived from synovial fluid in RA patients, multiple differentially expressed lncRNAs were discovered. Among them, ENST000000433825.1 is highly uniquely expressed in RA and significantly positively correlated with CRP, which may provide diagnostic and therapeutic biomarkers for RA.^176^

EV levels and content also showed significant differences in patients with multiple sclerosis (MS),^262^, ^263^, ^264^, ^265^, ^266^ allowing for monitoring of disease activity and prediction of disease progression.^264^ The neurologic and immunologic subclasses of EV contain AMBP, FIBB, GELS, IGHG4, and MBP, which provide information about the pathology of MS, reflect disease activity, and can differentiate between patients' disease phenotypes.^267^ Analysis of serum‐derived EV microRNAs also demonstrated that a variety of microRNAs were associated with MS recurrence and activity,^264^, ^268^, ^269^ and can be used to support personalized treatment decisions. Exosomes are also used in the diagnosis and monitoring of immune disorders such as multifocal leukoencephalopathy (PML),^270^ Sjögren's syndrome,^271^ autoimmune skin diseases,^272^ IgA neuropathies,^273^ adult myasthenia gravis,^274^ and inflammatory bowel disease.^275^, ^276^

In addition to the above diseases, exosomes also show the potential as noninvasive biomarkers in the diagnosis, prediction, and treatment of preeclampsia,^277^ corneal diseases,^278^ and transplant rejection.^279^ In summary, exosomes play an important role as biomarkers in the diagnosis, progression, and even efficacy monitoring of various diseases, which can help clinicians detect diseases earlier, evaluate efficacy, and provide a basis for individualized treatment of diseases.

## 4 EXOSOME THERAPY STRATEGIES

**FIGURE 3:**
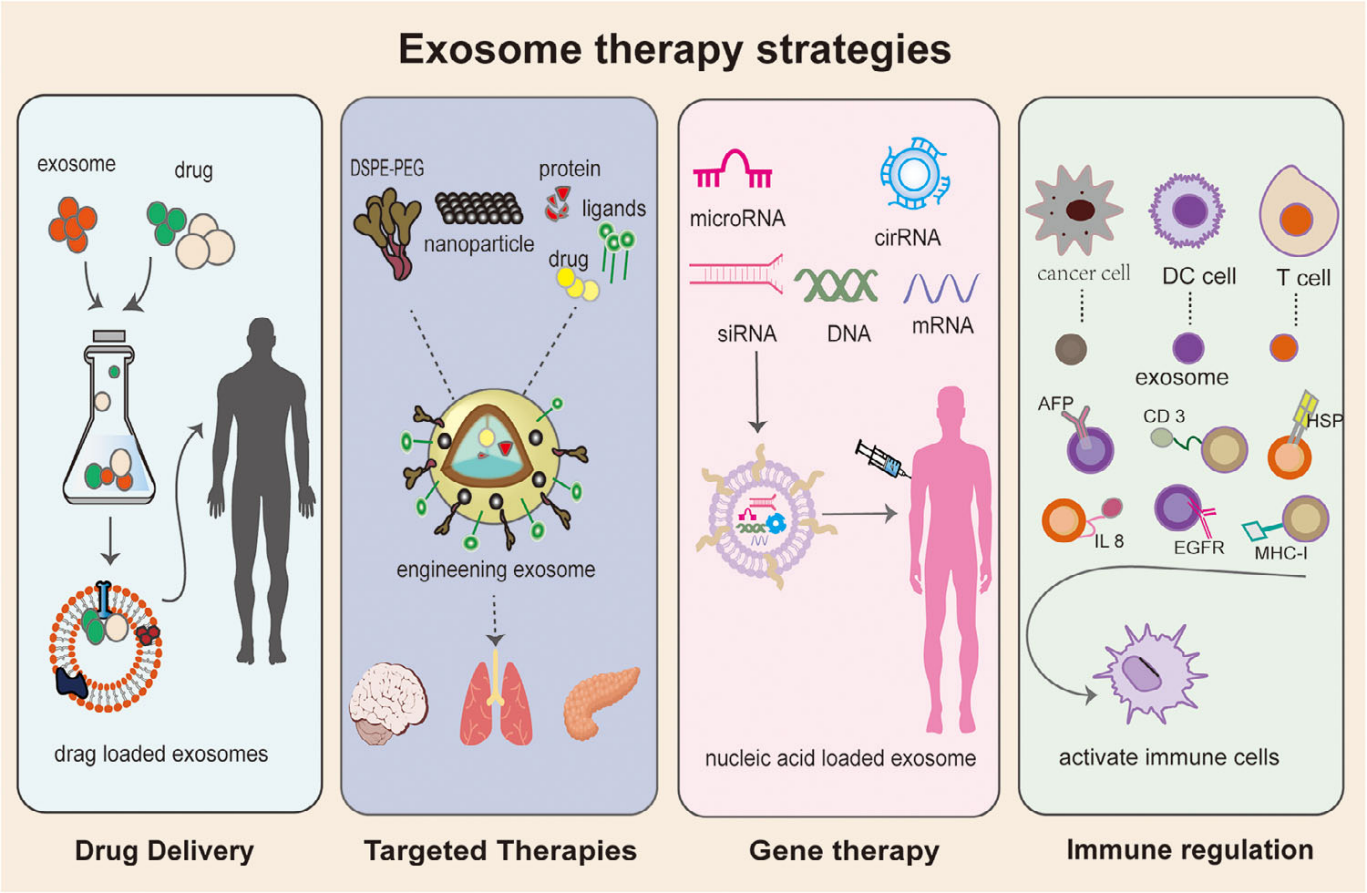
Therapeutic strategies of exosomes. As high‐quality delivery materials, exosomes can improve the precision of drug delivery by directly loading small molecule drugs. Modified exosomes, which can be loaded with drugs, proteins, lipids, and nucleic acids, not only enhance drug targeting, but their ability to safely present nucleic acids is also a highlight of gene therapy. In addition, exosomes derived from tumor cells and immune cells carry a variety of immune‐related molecules, which, after modification, can regulate immunity and be used in the treatment of immune diseases, immunotherapy of tumors, and the development of immunological vaccines (by Adobe Illustrator). HSP, heat shock protein; DC‐cell, dendritic cell. IL8, interleukin‐8.

Exosomes are nanoscale vesicular structures containing a variety of specific contents with the ability to target specific tissues, low immunogenicity, easy transport of hydrophilic or hydrophobic biomolecules, and the ability to cross biological barriers and penetrate tissue structures. This advantage allows exosomes to play multiple roles in disease treatment (Figure 3).

### 4.1 Drug delivery

For many years, research on drug presentation through nanoparticles (NPs) such as liposomes, micellar NPs, and superparamagnetic iron oxide NPs (SPION) has been going on, and some results have been achieved.^280^, ^281^ However, there are side effects such as early elimination, biological incompatibility, long‐term toxicity, and so on.^282^ Exosomes, as high‐quality delivery materials, have obvious advantages in terms of drug delivery capacity and toxicity.^283^ Nowadays, a large number of experiments have confirmed that exosomes can improve the precision of drug delivery and are efficient drug carriers without toxic side effects.

Back in 2015 researchers’ paclitaxel and adriamycin loaded into brain endothelial cell‐derived exosomes, and exosomal delivery of anticancer drugs in a zebrafish brain cancer model significantly reduced the fluorescence intensity and tumor growth markers of xenografted cancer cells, confirming the hypothesis that exosomes can deliver anticancer drugs to the BBB for the treatment of brain cancer.^284^ Qu et al.^285^ subsequently succeeded in delivering dopamine to the brain via exosomes in the blood, and in a mouse model of PD, exosomes loaded with dopamine showed better therapeutic efficacy and lower systemic toxicity than free dopamine after intravenous administration. Since then, several studies of exosomes as small molecule drug carriers loaded with chemotherapeutic agents have been reported, such as exosomal delivery of the chemotherapeutic agents adriamycin,^286^ paclitaxel,^287^, ^288^ DDP,^289^ and gemcitabine,^290^ which have been shown to enhance the cytotoxicity of the drugs on cancer cells and accelerate tumor regression in a variety of tumors. Exosomes loaded with curcumin^291^ and rosmarinic acid^292^ were also developed to enhance anti‐inflammatory activity. Loading brain‐derived neurotrophic factor (BDNF) in neural stem cell exosomes reduces the volume of ischemic cerebral infarction and improves neurological function.^293^ In addition to intravenous administration, in recent years EV has been found to help overcome the limitations of oral administration.^294^ For example, exosomes derived from grapefruit, loaded with methotrexate, are used to treat diseases associated with intestinal inflammation.^295^ Encapsulation of anthocyanidins into milk exosomes results in more potent antitumor effects without toxic side effects, providing an effective, cost‐efficient, and safe alternative to oral anthocyanidins administration.^296^ Hesperidin (HES), a flavonoid present in various citrus fruits, has excellent anticancer activity. Exo‐HES was formulated by loading it onto exosomes and showed better anticancer activity and oral bioavailability in a mouse model of melanoma, which could improve the therapeutic efficacy of HES in melanoma treatment.^297^

Of course, exosomes can also serve as delivery vehicles for therapeutic macromolecules. For example, binding trastuzumab (T‐DM1) to exosomes derived from HER2‐positive cancer cells may be carried by exosomes to other cancer cells, resulting in reduced viability of the recipient cells.^298^ Happily, exosomes can also enable gene therapy through nucleic acid presentation and genetically engineered specific target presentation. We will describe this in detail below.

### 4.2 Targeted therapy for EV

Multiple studies have shown that extracellular vesicles have natural targeting capabilities based on donor cells.^299^ Currently, techniques for improving specific targeting of extracellular vesicles can be divided into direct modification (direct loading of therapeutic cargoes such as genetic elements, biomolecules, and drugs into exosomes) and indirect modification (modification using physical and genetic techniques).^300^, ^301^ Decorated exosomes can enhance drug targeting, increase drug accumulation, and reduce off‐targeting, thereby improving efficacy and reducing side effects^302^ (Table 2).

**TABLE 2:** Genetically engineered exosomes as drug delivery systems.

Therapeutic cargo	Targeting ligand	Target cells	Function	References
siRNA targeting STAT3	Angiopep‐2	Glioma (U251)	Enhanced drug BBB penetration and targeting ability	^303^
miR‐588	C(RGDyk)	MB‐231 HEK‐293	Reshape immunosuppressive TME to enhance the antitumor effect	^304^
PTX	EGFR‐targeted peptides	H358 HCC827	Improving efficacy in EGFR‐positive tumors	^305^
PTX	AA	Murine lung cancer (3LL‐M27)	Improved normalization of blood vessels to make cancer sensitive to chemotherapy	^306^
Doxorubicin	iRGD	Breast cancer cells	Highly effective targeted DOX	^307^
Indocyanine green	Folic acid	Breast cancer cells	Increasing the efficacy of ultrasonodynamic therapy for breast cancer	^308^
siRNA targeting PD‐L1	RGD peptide	Glioma cell	strategy significantly improves GBM targeting efficiency as well as CD8 cytotoxic T‐cell activity	^309^
5‐Fluorouracil anti‐miRNA‐21	zHER affibody	Colorectal cancer (HCT‐116)	Targeted delivery of 5‐fu for increased efficacy	^310^
KGN	E7 peptide	SF‐MSCs	Cartilage regeneration	^311^
Curcumin	C(RGDyk)	Cerebral vascular endothelial cells	Reduce inflammation and inhibit apoptosis in ischemic stroke	^312^
Curcumin‐SPION	Neuropillin‐1‐targeted peptide	Glioma (U251)	Simultaneous diagnosis and treatment of glioma	^313^

Abbreviations: AA, aminoethylanisamide; KGN, Kartogenin; PTX, paclitaxel; STAT3, signal transducer and activator of transcription 3; SF‐MSCs, synovial fluid‐derived mesenchymal stem cells.

Direct exosome engineering allows the loading of various types of ligands onto exosome membranes through coupling and hydrophobic insertion.^314^ DSPE–PEG is a widely used module for anchoring targeting molecules to the exosome surface in lipid insertion.^315^ Exosomes modified with aminoethyl anis amide‐PEG (AA‐PEG) through DSPE–PEG insertion has been shown to target sigma receptors overexpressing lung cancers and can accumulate in large numbers in cancer cells, resulting in better therapeutic efficacy.^306^ Application of DSPE–PEG‐modified angiopep‐2‐EX to enhance BBB penetration and targeted drug delivery for the treatment of glioblastoma.^303^ In the study of triple‐negative breast cancer (TNBC), this technology was also used to modify the c(RGDyK) peptide on the surface of EV to deliver miR‐588, which can anchor CCL5 affected by the vicious circle of immune escape to reshape immunosuppressive TME to enhance the antitumor effect.^304^ The c(RGDyK) peptide also modifies EVs by chemical linkage. Engineered RGDyK‐conjugated exosomes (cRGD‐Exo) modify curcumin‐loaded MSC‐EVs, which can be targeted to diseased regions of the ischemic brain, leading to a strong inhibition of inflammatory responses and apoptosis in diseased regions.^312^ The same research team also confirmed that the engineered exosomes loaded with cholesterol‐modified miR‐210 (RGD exo: miR‐210) can increase the aggregation of miR‐210 in brain lesions, which is beneficial for the treatment of cerebral ischemia.^316^ Qi et al.^317^ used a receptor–ligand binding method to affix superparamagnetic magnetite colloidal nanocrystal clusters to the surface of exosomes for targeted drug delivery to tumors. Both in vivo and in vitro experiments showed that exosome‐based drug carriers displayed excellent in vivo targeting ability and cancer inhibition.^317^ Vandergriff et al.^318^ coupled cardiac homing peptide to exosome membranes via hydrophobic interaction. Engineered exosomes target the infarcted heart, reducing fibrosis and scar size and increasing cell proliferation and angiogenesis.^318^ Through direct modification, researchers have also created many exosomes with specific targeting properties, such as EV coupling with EGFR‐targeted peptides or anti‐EGFR nano‐antibodies to promote their accumulation in EGFR‐positive cancer cells, which can enable PTX delivery to significantly enhance drug efficacy in EGFR‐positive lung cancer.^319^ Jayasinghe et al.^305^ demonstrated that exosomes modified by enzyme engineering have a strong antigen‐specific targeting ability to efficiently and specifically target cells and deliver therapeutic drugs. Further in vivo experiments demonstrated that these engineered EVs were able to improve the biodistribution of encapsulated and conjugated drugs and significantly slow down cancer progression, both through enzymatic ligation of peptide drugs and exogenous loading of RNA.^305^

To achieve better targeting, indirect modification can also be achieved by genetically modifying cells that produce exosomes. This technology has been extensively studied in the treatment of tumors. The genetic modification of exosome‐producing cells is achieved by transfecting genes expressing targeted molecules (e.g., peptides, receptors, and antibodies), which fuse with extracellular vesicle membrane components (such as the C1C2 domain of ubiquitin, Lamp2b, and milk adhesive protein).^320^ Currently, LAMP‐2B is the most widely used exosome surface protein modification. Research has shown that the N‐terminus of LAMP‐2B is displayed on the surface of exosomes, which can attach targeted sequences and is the most widely used surface protein modification of exosomes.^321^ LAMP‐2B can be fused to specific binding peptides for tissue cell‐specific targeting. Using this technique, it was first observed that neuron‐specific RVG peptides fused to Lamp2b could specifically deliver siRNAs and induce knockdown of brain signaling specificity. However, EV targeting was affected by the degradation of the targeting peptide.^321^ Subsequently, in neuroblastoma studies, exosome‐targeted delivery was enhanced by the addition of a glycosylated glycan of the Lamp2b protein to prevent fusion protein degradation.^322^ In another experiment, the αv integrin‐specific (iRGD)peptide fused to exosome surface Lamp2b exhibits efficient targeting and delivery of DOX in αv integrin‐positive breast cancer.^323^ Similarly, RGD peptide and Lamp2b fusion‐modified exosome can target delivery of miR‐484, reprogramming the tumor vascular system and promoting chemosensitivity.^324^ The NSCLC homing peptide Tlyp‐1 coupled with Lamp2b selectively delivered siRNA to human NSCLC cells, thereby knocking down target genes and reducing the stemness of cancer stem cells.^325^

LAMP‐2B can also be genetically fused with targeting proteins or antibody fragments to display antibodies on exosomes.^320^ For example, the affinity molecule zHER, which targets HER2 binding, fuses with green fluorescent protein to the N‐terminus of Lamp2 on the surface of exosomes. zHER exosomes exhibit higher affinity and selectivity toward HCT‐116 colon cancer cells, allowing for more accurate delivery of 5‐FU and miR‐21i, thereby enabling the treatment of colorectal cancer.^326^ Chronic myeloid leukemia (CML) mother cells overexpress IL‐3 receptors (IL3‐R) on the cell surface, and fusion of IL‐3 receptors with EV Lamp2b can enhance EV accumulation in tumors. IL3‐EVs carrying imatinib and BCR‐ABL siRNA can target CML cells, effectively killing CML cells.^327^ In addition, the transmembrane proteins platelet‐derived growth factor receptor,^328^ the four‐transmembrane protein superfamily CD63/CD9/CD81^329^ and C1C2^330^ in exosomes have also been used for targeted modifications. For example, Apo‐A1 is coupled with CD63 to functionally deliver miRNA‐26a to SR‐B1 expressing HepG2 cells by utilizing purified exosomes from ApoA‐1 donor cells, thereby inhibiting their proliferation and migration.^331^ Exosomes can also be targeted by loading proteins. Hong et al.^332^ loaded PH20 hyaluronidase into exosomes inhibited tumor growth, increased T‐cell infiltration of tumors, and inhibited tumor growth better when coadministered with a chemotherapeutic agent (doxorubicin).

It can be seen that engineered EVs have achieved promising results in targeted drug delivery to specific cells and organs, as well as improving therapeutic efficacy.

### 4.3 Gene therapy

In recent years, exosomes as nonviral vectors have been at the forefront of gene therapy, particularly for applications in refractory diseases and regenerative medicines.^333^, ^334^

Exosomal nucleic acid delivery is one of the major highlights of gene therapy.^335^ For instance, HeLa cell‐derived exosomes deliver siRNAs for RAD51 and RAD52, which activate apoptosis in recipient cancer cells.^336^ Delivery of siRNA‐containing nanovesicles to Yes‐associated protein 1 (YAP) directs hepatocyte differentiation to normal hepatocyte‐like cells by activating downstream transcription factors, leading to tumor regression.^337^ Further studies have shown that siRNA‐loaded milk‐derived EV is a safe and achievable vehicle for oral nucleic acid therapies (e.g., RNA) in the management of intestinal diseases.^338^ Interestingly, it was found that exosome‐mediated siRNA successfully knocks out the therapeutic target BACE1 for AD through the BBB delivery through the BBB.^321^ Recently, it was found that exosomes of engineered chondrocytes, CAP‐Exo, which targets delivery of siRNA for MMP13, stimulated cartilage regeneration and attenuated cartilage degeneration.^339^ In the study of delivering miRNAs, researchers developed exosomes delivering miR‐204‐5p and found that they significantly inhibited the proliferation of tumor cells and increased sensitivity to chemotherapeutic drugs.^340^ Exosomal delivery of miR146a and miR‐200b ameliorates colitis^341^ and inhibits intestinal fibrosis.^342^ The genetically engineered exosomes are also highly efficient in delivering mRNA and have shown therapeutic effects in combination with other anticancer drugs.^343^ For example, exosomes loaded with peroxidase mRNA cross the BBB and improve the prognosis of Parkinson's disease in a mouse model of the disease.^344^ Researchers also loaded mRNA onto a charge‐reversed cationic exosome designed for the treatment of osteoarthritis, which overcame joint clearance and rapidly penetrated cartilage to promote cartilage regeneration.^345^ In another study, encapsulation of ECM α1 type I collagen (COL1A1) mRNA into EV effectively reduced collagen loss due to photoaging in a mouse model.^346^ Using a similar approach, exosomally encapsulated low‐density lipoprotein receptor mRNA can be used to treat familial hypercholesterolemia in mice.^347^

EVs can also be delivery vectors for CRISPR/Cas9 and participate in gene therapy. Xu et al.^348^ used chimeric antigen receptor‐modified EVs as a biologically safe delivery platform for the CRISPR/Cas9 system and showed that the CRISPR/Cas9 system was able to efficiently release anti‐MYC oncogenes in vivo and in vitro. Chen et al.^349^ constructed artificially designed extracellular vesicles for delivery of CRISPR/Cas9 targeting miR‐29b (EVs‐Cas9‐29b) could protect against muscle atrophy induced by dexamethasone (Dex), angiotensin II, and TNF‐α.

### 4.4 Modulating immunity

Exosomes can express a variety of immunomodulatory proteins on their surface, thus increasing their affinity and binding affinity to target receptors or ligands on immune cells and diseased cells, which can promote the formation of immune synapses and thus enhance the activation of the immune system.^350^ Studies have demonstrated that exosomes derived from tumor cells, CD4 T cells, DC cells, NK cells, macrophages (M1), and CAR‐T cells can directly or indirectly improve the immune response.^351^, ^352^, ^353^ The exosome derivatives produced after modification are more capable of enhancing immunomodulation and are widely used in tumor immunotherapy and cancer vaccine research. For example, IL‐18 and IL‐2 genetically modified TEX stimulate antitumor immune responses.^354^, ^355^ Antitumor immune responses are also induced by surface anchoring of superantigenic staphylococcal enterotoxin A.^356^

The efficacy of tumor immunotherapy depends largely on the sustained activation and expansion of tumor‐specific T cells, especially tumor‐infiltrating cytotoxic T lymphocytes (CTLs).^357^ The ability of exosomes to activate T cells is enhanced by the addition of adjuvants.^358^ HSP are highly enriched in TEX and have potent adjuvant capabilities. HSP treatment increasing the immunostimulatory activities of TEXs has been demonstrated in A20 lymphoma/leukemia cells.^359^ Epigenetic drug MS‐275 upregulates the expression of Hsp70, MHC‐I, and MICB in exosomes, which significantly enhances the cytotoxicity of NK cells.^360^ Expi293F cell‐derived exosomes were loaded with monoclonal antibodies against human T cell CD3 and EGFR, as well as immune checkpoint modulators, PD‐1 and OX40 ligand (OX40L). The resulting genetically engineered multifunctional immunomodulatory exosome (GEMINI‐Exos) not only redirects and activates T cells to kill EGFR‐positive TNBC cells but also induces potent anticancer immunity.^350^ Exosomes can also enhance immunity by regulating immunosuppression. Surface modification of bone marrow mesenchymal stem cell exosomes with oxaliplatin predrugs followed by delivery of galactaglutinin‐9 siRNA to PDAC tissues inhibits macrophage polarization, CTL recruitment, and downregulation of Tregs to stimulate antitumor immunity in the tumor.^361^

In the development of cancer vaccines, DC‐derived EVs (DEVs) are the most studied because of their ability to interact with CD4 and CD8+ T cells as well as NK cells to elicit an effective immune response.^362^ A personalized cancer vaccine based on recombinant adenovirus‐infected DC membranes genetically engineered to significantly improve antigen delivery to lymphoid organs and generate broad‐spectrum T‐cell responses.^363^ Exosomes prepared from ovalbumin (OVA)‐pulsed, activated DC were modified with anti‐CTLA‐4 antibody (EXO–OVA–mAb), which increased the migration of CD4 and CD8 T cells to the tumor site and increased the ratio of CTLs/Tregs in the microenvironment of the B16 melanoma tumor model.^364^ Munich et al.^365^ used mouse BMDC DEVs loaded with TNF, FasL, and TRAIL and observed that these vesicles were able to directly kill B16 tumor cells by promoting apoptosis and also induced NK cell activation and production of IFNγ.

In addition, the researchers have developed FAP genetically engineered tumor cell‐derived exosome‐like nanovesicles (eNVs‐FAP). As a tumor vaccine, eNVs‐FAP, which suppresses tumor growth by inducing a strong and specific CTL immune response directed against tumor cells and FAPCAF, as well as reprogramming immunosuppressive TMEs in models of colon, melanoma, lung, and breast cancer.^366^ In another, a prophylactic vaccine against ESC‐exo/granulocyte‐macrophage colony‐stimulating factor was developed, which inhibited the progression of metastatic lung tumors in mice.^367^

In conclusion, the exosome derivatives produced after processing are superior to native exosomes in modulating immunity and can be used both as novel and effective cancer vaccines and in immunotherapy.

## 5 CLINICAL APPLICATIONS AND CHALLENGES

Exosome‐based therapeutic strategies, many preclinical studies have demonstrated the broad significance of exosomes in the diagnosis, monitoring, prognostic assessment, vaccine development and treatment of diseases, and have led to the entry of exosomes into clinical studies for the diagnosis and treatment of diseases.

### 5.1 Clinical use as biomarkers in disease diagnosis and prognosis

Exosomes are now being translated to the clinic as diagnostic markers for cancer. Yoshioka et al.^368^ developed “ExoScreen,” an analytical technique for early detection of colorectal cancer, based on the fact that from raw blood samples from patients, EVs are trapped by CD147 (colorectal cancer‐specific antibody) and CD9 (exosomal membrane marker), and detected by photosensitizing beads. McKiernan et al.^369^ developed the “ExoDx Prostate (Intelliscore) EPI test” for the detection of prostate cancer, which is approved for clinical use by the Food and Drug Administration. This is based on the isolation of exosomal mRNA and the detection of PCA3 and ERG genes in a urine sample from the patient. On top of that, the results of several clinical trials using EV as a biomarker for diagnosis and disease surveillance have been published. For example, In a single‐center observational cohort study (NCT04928534), serum exosomes CHL1, KIF2A, miR‐1183, and miR‐297 were found to be potential biomarkers of CTE in patients with repetitive mild traumatic brain injury.^370^ A previous prospective, multicenter clinical utility study of the ExoDx Prostate test as a predictor of prognosis in high‐grade prostate cancer (NCT03235687) showed that the ExoDx Prostate (EPI) test had a significant impact on prostate biopsy decisions.^371^ The team recently reported interim results with 2.5 years of follow‐up, suggesting that prebiopsy EPI testing also leads to better outcomes when followed for 2.5 years after the initial biopsy decision‐making process.^372^ Another prospective study confirmed the efficacy of EV derived from adipocytes as an immediate biomarker for evaluating treatment response to nonalcoholic fatty liver disease.^373^ Mengfan's team demonstrated^374^ that complement proteins in plasma ADEs are associated with cognitive impairment in patients with obstructive sleep apnea (OSA) and can be used as a marker of cognitive impairment in OSA patients (ChiCTR1900021544). Another recent study on early diagnosis in patients with AD found that MiR‐125b‐1‐3p and miR‐451a have sufficient specificity and sensitivity for AD and are potential diagnostic biomarkers.^182^

Although EV has yielded good results in biomarkers, it also has some problems. As with conventional tumor markers, exosome markers can have false positive and false negative results. The contents secreted by normal cells may mask tumor secretion, and the same ones that are highly expressed in tumors may be positive in certain inflammatory conditions.^375^, ^376^ For example, miR‐21 is significantly expressed in glioblastoma but is also positively expressed in patients infected with Apache encephalitis virus infection.^377^ Coupled with the fact that the accuracy of the assay is correlated with the number of exosomes, the method of sampling and isolation of exosomes also puts limitations on clinical translation. To improve diagnostic accuracy, it is suggested that combining assays with multiple markers may provide a more precise diagnosis.^378^, ^379^

### 5.2 Therapy and efficacy of exosomes in clinical studies

Most of the ongoing clinical studies of exosomes for therapeutic use are in the early stages (phases I and II), with only a few in Phase III, covering a wide range of different disease indications, including cancer, cardiovascular disease, infectious diseases, and others (Table 3). According to a search on clinicaltrials.gov, 26 registered trials of exosome therapy have been completed, most of which unfortunately have not yet published their final results. The following is a description of the test for the published data.

**TABLE 3:** Clinical trials of exosomes registered at clinicaltrials.gov.

Application	Clinical setting	Stage	Start year	Source of exosome	Sponsor	Clinical trial number
Biomarker	Ocular muscle myasthenia gravis	NA	2023	Serum	First Affiliated Hospital of Jinan University	NCT05888558
	Immunotherapy in renal cell carcinoma	NA	2023	Blood and urine	Zhejiang Cancer Hospital	NCT05705583
	Locally advanced breast cancer	NA	2021	Serum	Samsung Medical Center	NCT05955521
	Gastrointestinal cancers	NA	2024	Blood	Beijing Friendship Hospital	NCT06278064
	Intrahepatic cholangiocarcinoma	NA	2023	Blood	City of Hope Medical Center	NCT06381648
Drug delivery	Inflammatory responses (several diseases)	1	2023	Engineered exosome	ILIAS Biologics Inc.	NCT05843799
	Inflammatory bowel disease	NA	2018	Ginger exosomes	University of Louisville	NCT04879810
	Homozygous familial hypercholesterolemia	1	2021	Plasma	Tang‐Du Hospital	NCT05043181
Therapy	Diabetic foot ulcers	2	2024	Purified Exosome Product	Rion Inc.	NCT06319287
	Acute ischemic stroke	1	2024	Pluripotent stem cell	Xuanwu Hospital, Beijing	NCT06138210
	Decompensated liver cirrhosis	2	2023	Mesenchymal Stem Cells	Research Institute for Gastroenterology and Liver Diseases	NCT05871463
	Refractory focal epilepsy	1	2023	Engineered exosome	Peking Union Medical College Hospital	NCT05886205
	COVID‐19 Acute respiratory distress syndrome	1 2	2023	Mesenchymal Stem Cell	AVEM HealthCare	NCT04798716
	Metastatic pancreas cancer	1	2021	Engineered exosome	M.D. Anderson Cancer Center	NCT03608631
	Degenerative meniscal injury	2	2022	Mesenchymal Stem Cell	Eskisehir Osmangazi University	NCT05261360
	Acute respiratory distress syndrome	3	2022	Bone marrow mesenchymal stem cell	Direct Biologics, LLC	NCT05354141
	Retinitis pigmentosa	2 3	2022	Mesenchymal stem cells	TC Erciyes University	NCT05413148

*Data sources*—clinical registration website.

Results of two phase I studies of DC‐derived exosomes for use as vaccines were published as early as 2005, two trials of DC‐derived exosomes (Dex) loaded with MAGE tumor antigens and MAGE 3 peptides in patients with unresectable lung cancers^380^ and melanoma,^381^ although one trial did not elicit an immune response, both trials confirmed the feasibility and safety of exosome administration. Also, in clinical trials of Dex modulation of immunity, investigators in two phase I trials in end‐stage cancers observed that the first generation of Dex (without IFN‐γ) could exert an NK cell effect. Since then with preclinical studies confirming that adjuvants (TLR4L or interferon (IFN) γ) enhance the stimulatory function of Dex on T‐cells using the treatment,^382^ the team went on to produce a second generation of Dex (IFN‐γ‐Dex), optimized for treatments aimed at boosting the immune response of both NK and T‐cells. This phase II clinical trial demonstrated that IFN‐γ‐DexDex, loaded with MHC class I and class II‐restricted cancer antigens, enhanced antitumor immunity in patients with advanced NSCLC.^383^

In a study of malignant pleural effusions, there was a significant reduction in tumor cells and reversal of TRC resistance after 7 consecutive days of intrapleural injections of exosomes loaded with DDP, compared with patients who received only DDP.^384^ The same results were subsequently obtained in another study of loaded MTX exosomes for the treatment of malignant pleural effusions.^385^ Based on the above, the investigators designed another randomized controlled clinical trial with a slightly larger sample to evaluate the safety and efficacy of intrapleural administration of MV–MTX in combination with pemetrexed and DDP chemotherapy for the treatment of malignant pleural effusion in advanced nonsquamous NSCLC. The results of the study showed that the amount of pleural effusion was significantly reduced in the MV–MTX group compared with the control group. At 1 year after treatment, the survival rate was 77.5% in the MV–MTX group and 59.0% in the placebo group, demonstrating that EVs can be used as an effective delivery vehicle for chemotherapeutic agents for cancer treatment.^386^

Due to the prevalence of COVID‐19 in previous years, researchers have also done a lot of research on introducing EVs for the treatment of COVID‐19. In a nonrandomized open‐label cohort study evaluating exosomes from allogeneic bone marrow mesenchymal stem cells (EXOFlo) for the treatment of severe COVID‐19, EXOFlo significantly improved symptoms, reduced mortality, and no adverse effects were observed within 72 h of treatment.^387^ Immediately following this, an RCT of ExoFlo infusion for the treatment of COVID‐19 respiratory failure yielded the same results and demonstrated the efficacy of a 15 mL dose of ExoFlo in COVID‐19‐induced asthma.^388^ Subsequently, researchers explored the safety and efficacy of aerosolized inhaled human adipose mesenchymal stem cell exosomes (haMSC‐Exos) in COVID‐19 patients. In this Phase IIa single‐arm, open‐label, interventional trial. After aerosol inhalation of haMSC‐Exos, all patients experienced varying degrees of regression of lung lesions. All COVID‐19 patients tolerated haMSC‐Exos aerosol therapy well.^389^

Exosomes have also yielded good results in studies of other diseases. Exosomes from mesenchymal stromal cells (MSC‐exo) administered as eye drops significantly alleviated dry eye associated with GVHD, in a prospective clinical trial.^390^ The first phase I/II clinical trial of three‐arm drug intervention has also released partial results regarding the safety and efficacy of allogeneic human adipose MSCs Exos (ahaMSCs Exos) in mild to moderate AD patients. In the medium dose group, the Alzheimer's Disease Assessment Scale Cognitive Scale (ADAS cog) score decreased by 2.33 at week 12.^391^ There have also been two randomized trials showing that adipose tissue stems cell‐derived exosomes (ASCEs) combined with microneedling/fractional CO_2_ lasers can have a synergistic effect on facial ageing^392^ and will have a significant improvement on atrophic acne scars.^393^

Building on the results of preclinical studies, scientists are currently conducting extensive clinical studies, which are briefly described below. In a study examining the ability of plant exosomes to deliver curcumin to normal and malignant colon tissues (NCT01294072), curcumin was loaded into plant exosomes and acted as a drug delivery system in normal and colon cancer tissues. Prior preclinical studies found that plant exosomes release exosomal particles that bind tightly to a variety of hydrophobic drugs, including curcumin, and are taken up by intestinal cells and intestinal immune cells.^394^ Chimeric exosomes iExosomes effectively used in preclinical studies were included in the clinical trial (NCT03608631). siRNA Kras G12D was loaded into exosomes derived from mesenchymal stromal cells and used as an anticancer drug for the treatment of patients with metastatic pancreatic cancer in this ongoing trial. Preclinical findings suggest that exosomes released from the placenta carry specific cargoes that lead to preeclampsia,^395^, ^396^ and by isolating these exosomes from maternal blood and placental tissues of patients diagnosed with preeclampsia and investigating their biochemical, cellular, and molecular mechanisms in animal models, the researchers hope to elucidate the key role that exosomal cargoes play in the development of preeclampsia and cardiovascular remodeling (NCT04154332). Several previous Phase I∖II studies have confirmed the utility of ExoFlo in respiratory distress syndrome. To further investigate the safety and efficacy of ExoFlo in the treatment of hospitalized patients with moderate to severe acute respiratory distress syndrome (ARDS), the investigators designed a Phase III, multicenter, randomized, double‐blind, placebo‐controlled trial (NCT05354141).

These clinical studies demonstrate the great possibilities of exosomes in medicine and provide an important basis for clinical translation.

### 5.3 Regulation and challenges of exosome therapy

The authoritative report from BCC Research in 2024 shows that the global market for exosome diagnostic, therapeutic, and research tools is expected to grow from $227.5 million in 2023 to $1.3 billion in 2028. Undoubtedly, exosomes have sparked a wave of biomedical research.

However, with the increase in research, the regulatory issues of exosome therapy have not been addressed. Current considerations include the following: (1) *Standardization of preparation and storage*. The preparation of exosomes varies from enterprise to enterprise in terms of purification, quantification storage methods, and so on, and there is a lack of mature industry standards. (2) *Scale‐up preparation*. Current cell culture and exosome purification methods limit the implementation of large‐scale production of exosomes.^397^ Although methods such as ultrafiltration, size exclusion chromatography, and aqueous two‐phase systems that are compatible with large‐scale production have been developed, there are no comparisons between them, and there is a lack of recommendations for regulated scale‐up preparation methods. (3) *Standardized control of exosome quality*. According to the different states of different parental cells, exosomes are highly heterogeneous in carrying nucleic acids and proteins. Standardized quality control is the guarantee for the clinical application of exosomes. (4) *Regulatory gaps*. Currently, there is no standardized set of violations for using extracellular vesicles to serve supervision.

Of course, exosomes also face various challenges from preclinical to market. Due to the small size and complex composition of EVs, detecting and evaluating changes in the quality of electric vehicle products is challenging. The production of EVs, especially the selection and modification of donor cells to produce exosomes, is hampered by low yields, high prices, and difficulties in industrial production. How to produce exosomes quickly, economically, and in large quantities is a major challenge for clinical translation. Exosomes have become a key element in the field of therapeutic delivery, but the large number of natural components carried by exosomes themselves makes cargo loading very complex and restricted, and it has been a challenge to develop exosome engineering techniques for efficient and reproducible loading of therapeutics into EVs. Exosomes are highly heterogeneous and separating homogeneous exosomes from heterogeneous exosomal motifs is technically challenging. Quantifying the exact amount of EV per unit therapeutically remains difficult, and finding a strategy that allows precise control of exosome content is also challenging for scientists.

For these challenges, in the future, interdisciplinary teams from science, chemistry, engineering, and medicine will collaborate in what is expected to be a transformation of exosomes from the laboratory to clinical practice.

### 5.4 Prospects and potential R&D directions

With the development of biotechnology and an in‐depth understanding of exosome functions, research on exosome technology has made great progress. The emerging magnetic NP capture technology has effectively improved the purity and efficiency of exosome isolation. Microfluidic microarray technology enables richer and more precise biological information to be obtained from individual exosomes. In addition, the development of Surface Enhanced Raman Spectroscopy sensors, immunomagnetic bead detection technology, and fluorescence technology has improved detection sensitivity and specificity.^398^ We have reason to believe that exosomes will revolutionize the medical field and promote the advancement of human health.

Exosomes will play an increasingly important role in the future of medical technology. It not only improves the precision and efficiency of drug delivery but also aids in the early detection and treatment of disease. With the deepening of scientists' understanding of exosomes, exosomes can also develop many potential research directions. First, current exosome research mainly focuses on plant and animal sources, and little is involved in microbe‐derived exosomes. Can these bacterial and viral‐derived exosomes, which carry information from their parents and are modified, play a beneficial role in infectious diseases, tumors, and other intractable diseases? Second, exosomes are mainly administered intravenously, but can also be sprayed and given orally. With the development of biomaterials, it will be possible to develop customized drug delivery methods for specific diseases by wrapping exosomes with special materials. Furthermore, exosomes have achieved some results in vaccine research. In the treatment of tumors, can synergistic effects be achieved by the combined application of exosome vaccines with radiotherapy and immunotherapy? Finally, Exosomal contents are complex, and the effects of many RNAs and DNAs on the body through cellular communication are not yet fully understood. Deepening research in this area may open up new horizons in the treatment of exosomes.

## 6 SUMMARY

Exosomes, as nanoscale vesicles, are one of the most effective means of intercellular communication, which regulates the function of receptor cells. Through extensive research, researchers have found that exosomes play an important role in disease treatment. Exosomes can carry information from parental cells and exist in various body fluids, making them the most promising biomarkers for liquid biopsy. It can be used for early diagnosis of diseases, and more importantly, it has been found to serve as an indicator for monitoring drug efficacy, disease progression, and to detect the sensitivity of diseases to certain drugs. More recently, this technology set has been combined with machine learning and artificial intelligence (AI), which has had a significant impact on the fast‐reading screening of diseases, especially the diagnosis of cancer.^399^ Since exosomes have a natural advantage over other NPs, they are also a favorable contender for drug delivery. EVs can accurately transport a variety of molecules, proteins, and even nucleic acids to targeted cells. Most encouragingly, EVs show a broad biodistribution that crosses the BBB and holds promise for the treatment of CNS diseases. The exosomes secreted by some stem cells and immune cells have an indispensable role in tissue repair and immune response. In recent years exosomes have gained attention in tissue regeneration, tumor immunotherapy, and immune vaccine development, and some results have been achieved. Especially with genetically engineered exosomes, researchers have seen encouraging results in drug delivery, targeted therapy, and immune regulation. Next the combined application of exosomes and 3D printing has also brought new hope in tissue repair.

With the continuous progress of medical science and technology, the diagnosis and treatment of diseases have entered the era of precision medicine. Exosomes, as the most emerging molecules, will have a great role in promoting individualized and precise treatment. Exosomes as molecular markers can achieve precise detection of disease‐causing genes, and through the mediation of targeting and gene therapy, precise treatment of diseases can be achieved, and the development of nano‐vaccines opens up a new way of personalized immune regulation. In the future, the close integration of exosomes with emerging fields such as AI and bioengineering, will help to create diagnostic models for identifying specific diseases and new personalized treatment options. Exosome research and applications will revolutionize the field of medicine.

## AUTHOR CONTRIBUTIONS

*Writing—ideas, original drafts, graphic preparation, and literature searches*: Chuan Xu. *Writing—original draft*: Chaoyang Jiang. *Writing—original draft and funding acquisition*: Zhihui Li. *Writing—original draft*: Hui Gao. *Writing—original draft*: Jing Xian. *Writing—literature search*: Wenyan Guo. *Writing—literature search*: Dan He. *Writing—Review*: Xingchen Peng. *Review*: Daijun Zhou. *Writing—review and editing*: Dong Li. All authors have read and approved the final manuscript.

## CONFLICT OF INTEREST STATEMENT

The authors declare no conflict of interest.

## ETHICS STATEMENT

Not applicable.

## Data Availability

Not applicable.
